# Symptoms and sleep characteristics of tic disorder children with allergic diseases: a case–control study

**DOI:** 10.3389/fped.2025.1573463

**Published:** 2025-09-30

**Authors:** Zhang Panpan, Liu Yang, Wang Na, Ma Tao, Wang Jinyuan, Zhang Guixiang, Cui Yifan

**Affiliations:** ^1^ Department of Pediatrics, Dalian Municipal Women and Children’s Medical Center (Group), Dalian, Liaoning, China; ^2^ Dalian Medical University, Dalian, Liaoning, China; ^3^ Department of Critical Care Medicine, The First Hospital of Yulin, Yulin, Shaanxi, China

**Keywords:** tic disorder, allergy, Yale Global Tic Severity Scale, children’s sleep habits questionnaire, relationship

## Abstract

**Introduction:**

Recent studies have shown a close relationship between tic disorder (TD) and allergic diseases in children. Allergic diseases also have a significant impact on children's sleep. Regrettably, it remains unclear whether TD children with comorbid allergic diseases exhibit distinct symptoms and sleep characteristics.

**Objective:**

This study aimed to explore the symptoms and sleep characteristics of TD children with allergic diseases.

**Methods:**

This was a case–control study involving 242 TD children (aged 3–14 years), of whom 168 had allergic diseases and 74 did not have allergic diseases. General information and allergy histories were recorded for all participants. The Yale Global Tic Severity Scale (YGTSS) was used to assess TD symptoms. All guardians of TD children were required to complete the Children's Sleep Habits Questionnaire (CSHQ).

**Results:**

Compared with the group of TD children without allergic diseases, the comorbid allergic disease group had significantly higher impairment scale scores and total tic scores on the YGTSS (all *p* < 0.05). Parasomnias score, sleep-disordered breathing score, and CSHQ total score were also significantly higher in the TD group with combined allergic diseases (all *p* < 0.05). Further analyses revealed no significant difference in TD symptoms and sleep between groups based on the number of allergic diseases and control of allergic diseases (all *p* > 0.05). However, significant differences in TD symptoms and sleep occurred based on the type of allergic disease. Among them, the impairment scale score, total tic score, and sleep-disordered breathing score of the allergic rhinitis group were significantly increased (all *p* < 0.05); in the allergic conjunctivitis group, the total motor score and total tic score increased significantly, and the daytime sleepiness score decreased significantly (all *p* < 0.05). In addition, we found a correlation between the YGTSS and CSHQ scores.

**Conclusion:**

This study found that TD children with allergic diseases exhibited more severe clinical symptoms and higher CSHQ total scores. The clinical and sleep changes are particularly significant in TD children with different types of allergic diseases, especially allergic rhinitis and allergic conjunctivitis.

## 1 Introduction

Tic disorder (TD) is characterized by abrupt, rapid, recurrent, non-rhythmic motor and/or vocal tics, either single or multiple times, typically emerging at 5–6 years old (1). TD is classified into three types: Tourette's syndrome (TS), chronic motor or vocal tic disorders (CTD), and provisional tic disorders (PTD), based on their specific traits and the disease's advancement (2). In recent years, the incidence of TD has increased (3). Research by the CDC, utilizing parent-reported data, revealed that 0.3% (1 in 333) of children aged 3–17 in the USA have been diagnosed with TS (4). The general occurrence of TD among Chinese children varies between 1.04% and 2.98% (5, 6). Children with TD often present with one or more comorbidities, of which sleep problems are common but often overlooked clinically (7, 8).

A study showed a significant occurrence of sleep problems in children with TD, varying between 9.7% and 80.4%, as measured by subjective methods such as parent-reported questionnaires (9). In recent years, a growing body of evidence suggests that there is a complex bidirectional causal relationship between TD and sleep problems and that sleep problems can exacerbate the difficulty of treating TD throughout the lifespan of affected children, which is accompanied by a certain degree of growth retardation, increased cardiovascular burden, and risk of immune disorders. Additionally, sleep problems can be exacerbated in children with pre-existing sleep disorders due to the presence of TD (10–13).

Concurrently, various studies have recently found a strong link between TD and allergic conditions in children. In 1984, Bruun (14) noted that TS symptoms are usually associated with seasonal allergic reactions or the ingestion of food allergens. In 1985, Finegold (15) observed that the symptoms of TS patients might resemble or coexist with allergic conditions. In 1997, Kim et al. (16) proposed that certain foods might play a role in TS by enhancing the production of specific neurotransmitters. In 2011, Chang et al. (17) revealed a notable link between TS and allergic conditions, indicating that individuals with allergic rhinitis (AR) faced a twofold risk of TS (adjusted OR = 2.18). The adjusted ORs for allergic asthma (AA), atopic dermatitis (AD), and allergic conjunctivitis (AC) were 1.82, 1.61, and 1.33, respectively. In 2022, a meta-analysis strongly suggested a link between TD and allergic conditions (18). These findings indicate that TD is strongly associated with allergic diseases. In the same year, Chang et al. (19) revealed a link between allergic conditions and the onset of TD in children, noting that AC was most closely related to TD, followed by eczema and AR. They also observed that over 80% of the allergic conditions occurred before the onset of TD.

Allergic diseases have a significant impact on children's sleep. Studies indicate that sleep problems are more prevalent among children with AD than in adults. Children with AD experience difficulties in falling asleep, frequent nocturnal awakenings, and heightened daytime sleepiness (20). AR is a key risk element for habitual snoring in children, with its severity showing a significant and distinct correlation with pediatric obstructive sleep apnea (OSA) (21). Children with AA frequently exhibit symptoms such as insomnia, subpar sleep quality, difficulty initiating sleep, and sleep disturbances (22). The impact of allergic diseases on sleep is not only limited to physical symptoms but also affects psychological health. Allergic diseases and sleep problems are associated with increased psychological distress in children, similar to what is observed in TD (23).

Based on the associations found in previous investigations, we expect that TD children with comorbid allergic diseases may exhibit more severe clinical symptoms and a higher incidence of sleep problems. Regrettably, it remains unclear whether TD children with comorbid allergic diseases exhibit distinct symptoms and sleep characteristics. Therefore, this study aimed to explore the symptoms and sleep characteristics of TD children with allergic diseases.

## 2 Methods

### 2.1 Study design

The Ethics Committee of Dalian Municipal Women and Children's Medical Center (Group) at Dalian Medical University approved this cross-sectional study (FEJT-KY-2024-08). Subjects were recruited between October 2023 and October 2024 in the Department of Child Health at Dalian Municipal Women and Children's Medical Center (Group). All participants’ parents or guardians provided written agreement, and this study adhered to the ethical guidelines outlined in the Declaration of Helsinki.

### 2.2 Study population

The following section outlines the diagnostic criteria and the inclusion/exclusion criteria for participants in this clinical study. In accordance with the fifth edition of the Diagnostic and Statistical Manual of Mental Disorders (DSM-5), the diagnosis of TD in children was performed by specialists in child development and behavior. These professionals collected medical history through semi-structured interviews and integrated these data with clinical observations obtained during examinations. They used an electroencephalogram and other tests to rule out other diseases before confirming a TD diagnosis (24). Experienced experts in child development and behavior assessed the intensity of TD using the Yale Global Tic Severity Scale (YGTSS). All guardians of children with TD completed the Children's Sleep Habits Questionnaire (CSHQ). To reduce reporter bias, the guardians of TD children were strictly screened and required to accompany their children ≥5 days a week before completing the forms. A researcher was available to explain to the guardians the requirements for completing the questionnaire before they filled and to answer their questions at any time during the completion process. Specific information about allergic diseases was recorded by an allergy specialist, mainly including the type, number, and control of allergic diseases. This information was obtained promptly after the child was diagnosed with TD by filling in paper forms and semi-structured interviews.

Inclusion criteria for children with TD with allergic conditions were (a) a formal clinical diagnosis of TD; (b) age between 4 and 14 years; (c) a history of allergic reactions within the past year; (d) a previous diagnosis of allergic diseases. Inclusion criteria for children with TD without allergies were (a) a formal clinical diagnosis of TD; (b) age between 4 and 14 years; (c) no history of allergic reactions; and (d) no previous diagnosis of allergic disease.

Exclusion criteria for all participants were (a) aged <4 or >14 years; (b) presence of neurological conditions affecting central function (e.g., narcolepsy, epilepsy, autism spectrum disorder, and intellectual disability) or other significant mental health issues requiring hospitalization (e.g., depression and anxiety); (c) any severe health condition, such as inflammatory bowel disease, a history of cancer, diabetes, and liver or kidney disease; (d) ongoing use of any medications; and (e) incomplete case information. These exclusion criteria were mainly based on semi-structured interviews combined with case system screening.

### 2.3 Study measures

#### 2.3.1 History of allergy

Diagnosis of allergic diseases was performed by specialized pediatric allergists. Food allergy (FA) diagnosis was based on the EAACI food allergy and anaphylaxis guidelines (25), AR diagnosis relied on the ARIA guidelines (26), AA diagnosis according to criteria described by Global Initiative for Asthma (GINA) guideline, AD diagnosis according to consensus criteria of the Chinese Academy of Dermatology, and AC diagnosis according to classical descriptions (27). Each child with allergic disease had a positive skin prick test (≥3 mm) or allergen-specific IgE ≥0.35 IU/mL.

All children's allergic information was recorded in a timely manner after TD was diagnosed, including the type, number, and control of allergic diseases. These records were derived from the medical history provided by the parents and the hospital medical record system. The control of allergic diseases was categorized as poor, partial, and complete according to the clinical manifestations in the past month (28, 29).

#### 2.3.2 YGTSS

During clinical consultations, child development and behavior specialists use the Yale Global Tic Severity Scale (YGTSS), a reliable and valid tool, to gauge the intensity of tics experienced in the preceding week (30, 31). The YGTSS includes 40 possible twitch checklists, categorized into motor tic and phonic tic. The tics experienced in the previous week were evaluated using a five-point scale (number, frequency, intensity, complexity, and inference), whereas motor and phonic tics were assessed independently. The YGTSS records three levels of tic intensity: total motor (0–25), total phonic (0–25), and a scale for impairment ranging from 0 to 50. The total motor, total phonic, and impairment scale scores were added together to generate a total tic score.

#### 2.3.3 CSHQ

A modified version of the CSHQ was used in this study. The CSHQ is a retrospective, 45-item parent-reported questionnaire that has been widely used to examine sleep behaviors, including sleep time and sleep quality, where sleep time refers to nighttime sleep (32). Parents were asked to remember the sleeping behaviors of their children during a standard recent month. Items are assessed on a tripartite scale: “usually” indicates happening 5–7 times weekly; “sometimes” denotes 2–4 times weekly; and “rarely” represents 0–1 times weekly. Certain item scores will be inverted and require conversion for subsequent computations. Conceptually, the CSHQ is segmented into eight distinct subscales, each representing a different sleep aspect: bedtime resistance, sleep anxiety, sleep onset delay, parasomnias, sleep duration, sleep-disordered breathing, daytime sleepiness, and night wakings. Among them, sleep duration refers to irregular sleep duration, not nighttime sleep. The sum of the scores across the eight subscales yields the CSHQ total score, which reflects the overall sleep quality. Higher scores indicate poorer sleep quality, and a CSHQ total score above 41 points was considered indicative of poor sleep quality.

### 2.4 Statistical analyses

Data analysis was conducted using SPSS software, version 26.0. Continuous variables were presented as mean ± SD. Comparisons between the two groups were conducted via the Student’s *t*-test or Wilcoxon signed-rank test, while ANOVA or Kruskal–Wallis H test was used for multigroup comparisons. Categorical data were reported as *n* (%), and the *χ*^2^ test or Fisher's exact probability test was employed for comparing between groups. Spearman’s rank correlation was used to analyze the relationship between YGTSS and CSHQ scores. *p* < 0.05 was considered statistically significant.

## 3 Results

### 3.1 Characteristics of all TD participants

This study encompassed 242 children aged between 3 and 14 years diagnosed with TD, of whom 168 had allergic diseases and 74 did not. This study's clinical demographic characteristics are listed in Table 1. Basic characteristics such as age, males, body mass index (BMI), maternal smoking and drinking during pregnancy, birth history, feeding history, and the presence of siblings exhibited no statistical difference between the groups (all *p* > 0.05). However, TD children with comorbid allergic disease had significantly higher electronic screen exposure and paternal and maternal allergy histories compared with those without allergic diseases (all *p* < 0.05).

**Table 1:** Characteristics of all TD participants.

Subject (*n*)	TD + allergy group, *N* = 168	TD + no allergy group, *N* = 74
Age (years, mean ± SD)	7.49 ± 2.43	7.99 ± 2.55
BMI (kg/m^2^, mean ± SD)	17.97 ± 4.67	19.09 ± 7.04
Male, *n* (%)	135 (80.36)	55 (74.32)
Maternal smoking during pregnancy, *n* (%)∼	1 (0.01)	1 (0.01)
Maternal drinking during pregnancy, *n* (%)	2 (1.19)	0 (0.00)
Term birth, *n* (%)		
<37 weeks	11 (6.55)	4 (5.41)
37–42 weeks	150 (89.29)	68 (91.89)
>42 weeks	7 (4.17)	2 (2.70)
Cesarean, *n* (%)	74 (44.05)	37 (50.00)
Feeding style at least 6 months, *n* (%)		
Breast feeding	92 (54.76)	40 (54.05)
Artificial feeding	13 (7.74)	11 (14.86)
Mixed feeding	63 (37.50)	23 (31.08)
Electronic screen exposure >30 min/day, *n* (%)	103 (61.31)	35 (47.30)*
Maternal allergy, *n* (%)	72 (42.86)	5 (6.76)***
Paternal allergy, *n* (%)	59 (35.12)	9 (12.16)***

TD, tic disorder; BMI, body mass index; maternal smoking during pregnancy, ≥20 cigarettes per day or every other day; maternal drinking during pregnancy, ≥2 g of alcohol per day or every other day.

* There is a statistical difference between the two groups, *p* < 0.05.

*** There is a statistical difference between the two groups, *p* < 0.001.

### 3.2 Types of TD and YGTSS score in TD children with allergy

Table 2 displays the comparative outcomes of types and YGTSS between the TD combined allergy and without allergy groups. TD types were not significantly different between the two groups (*p* > 0.05). Although total motor score and total phonic score showed a trend toward higher scores in the comorbid allergic disease group, no statistical differences were observed between the two groups (all *p* > 0.05). In contrast, impairment scale score and total tic score in the comorbid allergic disease group were significantly higher than those in the group without allergy (all *p* < 0.05).

**Table 2:** Types and YGTSS in children with TD between groups.

TD type		TD + allergy group, *N* = 168	TD + no allergy group, *N* = 74	Statistics
Types of TD, *n* (%)	PTD	98 (58.33)	45 (60.81)	
	CTD	30 (17.86)	19 (25.68)	*χ*² = 4.240*, p* = 0.120
	TS	40 (23.81)	10 (13.51)	
YGTSS (mean ± SD)	Total phonic score	4.77 ± 4.60	4.07 ± 5.05	*Z* = −1.411, *p* = 0.158
	Total motor score	9.29 ± 3.96	9.08 ± 3.82	*Z* = −1.021, *p* = 0.307
	Impairment scale score	14.73 ± 6.86	11.35 ± 4.78	*Z* = −3.765, *p* < 0.001***
	Total tic score	28.79 ± 10.05	24.50 ± 8.35	*Z* = −3.185, *p* < 0.001***

YGTSS, Yale Global Tic Severity Scale; TD, tic disorder; PTD, provisional tic disorders; CTD, chronic motor or vocal tic disorders; TS, Tourette's syndrome.

*** There is a statistical difference between the two groups, *p* < 0.001.

### 3.3 Sleep problems in TD combined allergy

By comparison, it was found that parasomnia score, sleep-disordered breathing score, and CSHQ total score of the TD combined allergy group were significantly higher than those of the group without allergy (all *p* < 0.05). Hours of sleep per night and the remaining CSHQ subscales showed no notable statistical differences (all *p* > 0.05) (Table 3).

**Table 3:** CSHQ scores in children with TD between groups.

CSHQ	TD + allergy group, *N* = 168	TD + no allergy group, *N* = 74	Statistics
Hours of sleep per night (mean ± SD)	9.43 ± 0.79	9.51 ± 0.85	*Z* = −0.560, *p* = 0.575
Bedtime resistance (mean ± SD)	11.05 ± 3.04	10.51 ± 3.12	*Z* = −1.105, *p* = 0.269
Sleep onset delay (mean ± SD)	1.55 ± 0.66	1.43 ± 0.64	*Z* = −1.456, *p* = 0.145
Sleep duration (mean ± SD)	4.22 ± 1.40	4.15 ± 1.37	*Z* = −0.428, *p* = 0.669
Sleep anxiety (mean ± SD)	7.29 ± 2.27	7.05 ± 2.18	*Z* = −0.781, *p* = 0.435
Night wakings (mean ± SD)	3.83 ± 1.16	3.62 ± 0.93	*Z* = −1.199, *p* = 0.230
Parasomnias (mean ± SD)	8.90 ± 1.79	8.35 ± 1.45	*Z* = −2.153, *p* = 0.031*
Sleep-disordered breathing (mean ± SD)	3.65 ± 0.88	3.39 ± 0.76	*Z* = −2.536, *p* = 0.011*
Daytime sleepiness (mean ± SD)	13.36 ± 3.17	12.85 ± 2.74	*Z* = −1.118, *p* = 0.264
Total score (mean ± SD)	53.85 ± 8.25	51.36 ± 6.85	*Z* = −2.253, *p* = 0.024*

TD, tic disorder; CSHQ, children's sleep habits questionnaire.

* There is a statistical difference between the two groups, *p* < 0.05.

### 3.4 The timeline for the onset of TD and allergic diseases

**Figure 1:**
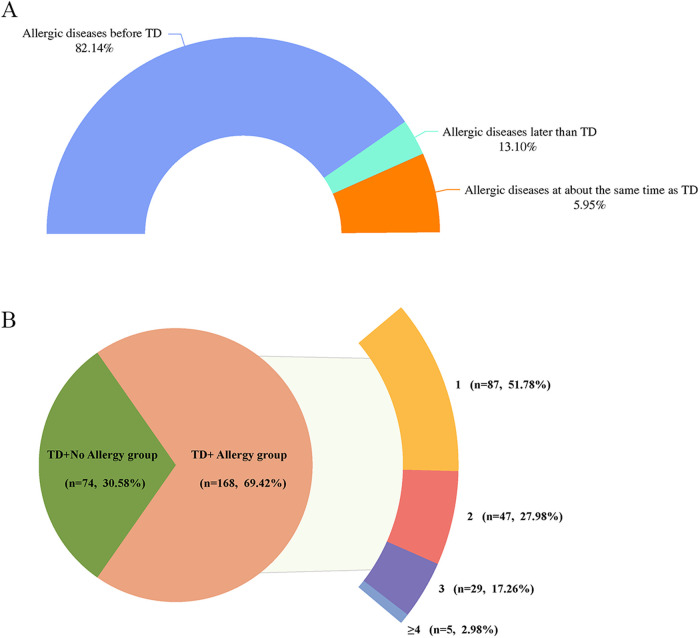
**(A)** Temporal distribution of onset of TD and allergic diseases. **(B)** Distribution of the number of allergic diseases among children with TD. TD, tic disorder; 1, combined with one allergic disease; 2, combined with two allergic diseases; 3, combined with three allergic diseases; ≥4, combined with at least four allergic diseases.

Among the children with TD combined allergic diseases, 82.14% had allergic diseases before TD, 13.10% had allergic diseases around the same time as TD, and 5.95% developed allergic diseases later than TD (Figure 1A).

### 3.5 Relationship between TD and the number of allergic diseases

Of the 168 children with TD who had a combination of allergic diseases, 87 (51.78%) had one allergic disease, 47 (27.98%) had two allergic diseases, 29 (17.26%) had three allergic diseases, and 5 (2.98%) had at least four allergic diseases (Figure 1B). Further analyses showed no statistically significant differences between TD types and YGTSS and CSHQ scores across groups based on the number of allergic diseases (all *p* > 0.05) (Supplementary Table 1).

### 3.6 Relationship between TD and the types of allergic disease

**Figure 2:**
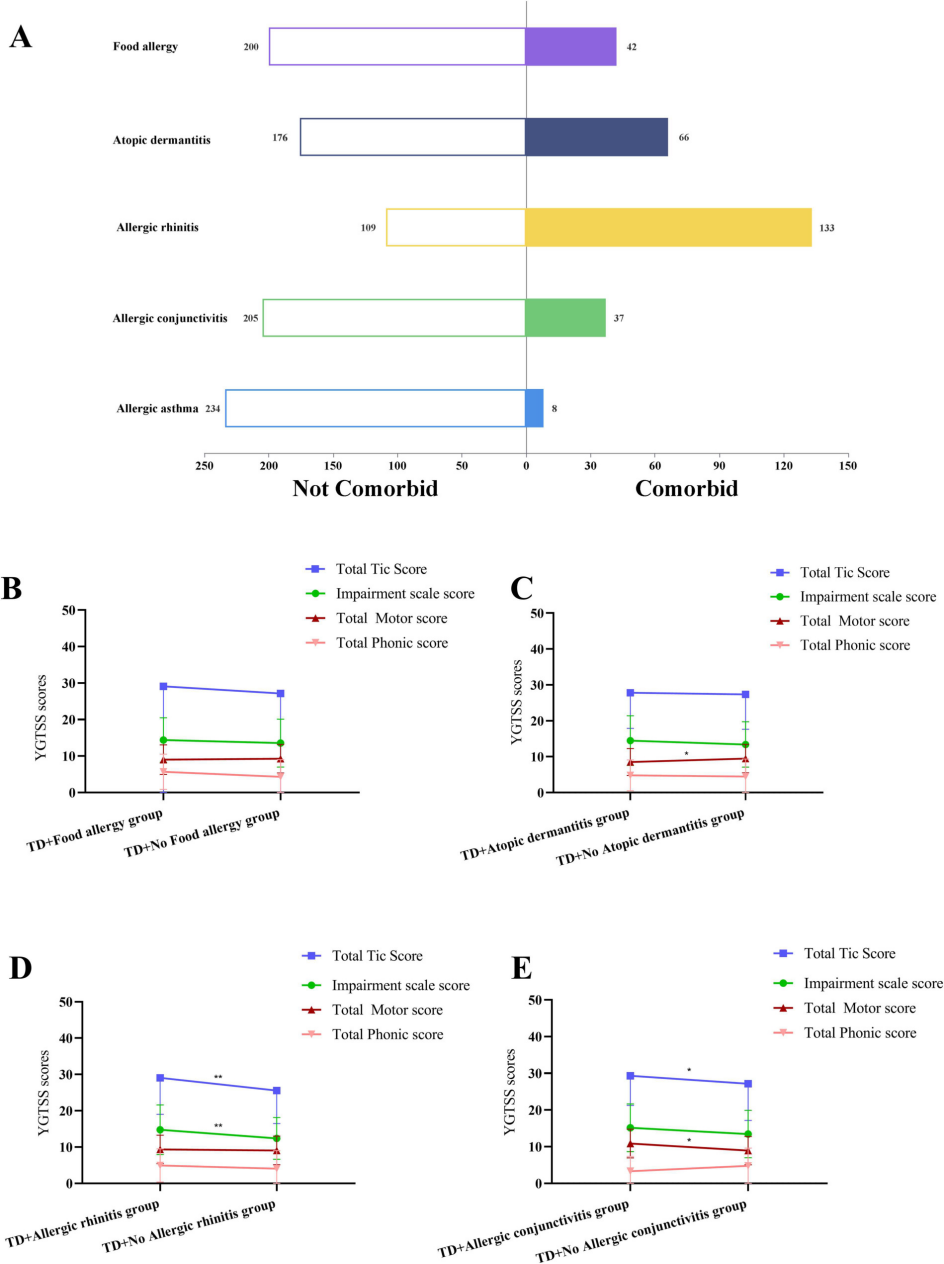
**(A)** Distribution of different types of allergic diseases in children with tic disorder. **(B)** Effect of combined food allergy on YGTSS scores in TD children. **(C)** Effect of combined atopic dermatitis on YGTSS scores in TD children. **(D)** Effect of combined allergic rhinitis on YGTSS scores in TD children. **(E)** Effect of combined allergic conjunctivitis on YGTSS scores in TD children. TD, tic disorder; YGTSS, Yale Global Tic Severity Scale; *There is a statistical difference between the two groups, *p* < 0.05. **There is a statistical difference between the two groups, *p* < 0.01.

An analysis of the types of allergic diseases in all TD children showed that 42 children with FA accounted for 17.36%; 66 children with AD accounted for 27.27%; 133 with AR accounted for 54.96%; 37 with AC accounted for 15.29%; and 8 with AA accounted for 3.31% of all children with TD. The distribution was shown in Figure 2A.

Next, TD children with different allergic disease types were analyzed one by one. Firstly, compared with TD children without FA, no notable statistically significant differences were observed in TD types and YGTSS and CSHQ scores in the combined FA group (all *p* > 0.05) (Figure 2B) (Supplementary Table 2).

Secondly, compared with the TD combined AD group, the total motor score was significantly higher in the TD without AD group (*p* < 0.05) (Figure 2C). The sleep duration score was significantly higher in the group without AD (*p* < 0.05). No statistical differences were seen in the remaining indicators (all *p* > 0.05) (Supplementary Table 3).

Thirdly, compared with TD children without AR, the impairment scale score and total tic score of YGTSS were significantly higher in the combined AR group (all *p* < 0.01) (Figure 2D). The sleep-disordered breathing score was significantly higher in the combined AR group (*p* < 0.001). No statistical differences were seen in the remaining indicators (all *p* > 0.05) (Supplementary Table 4).

Finally, compared with TD children without AC, total motor score and total tic score of YGTSS were significantly higher in the combined AC group (all *p* < 0.05) (Figure 2E). The daytime sleepiness score of CSHQ was significantly higher in the combined AC group (*p* < 0.05). No statistical differences were seen in the remaining indicators (all *p* > 0.05) (Supplementary Table 5).

### 3.7 Relationship between TD and the control of allergic disease

Based on the control of allergic symptoms in the last month, all children with comorbid allergic diseases were categorized as completely controlled (*n* = 102), partially controlled (*n* = 54), and poorly controlled (*n* = 12). Further findings showed that TD type and YGTSS and CSHQ scores showed no statistically significant differences between different control groups (all *p* > 0.05) (Supplementary Table 6).

### 3.8 YGTSS scores are associated with CSHQ in TD children

**Figure 3:**
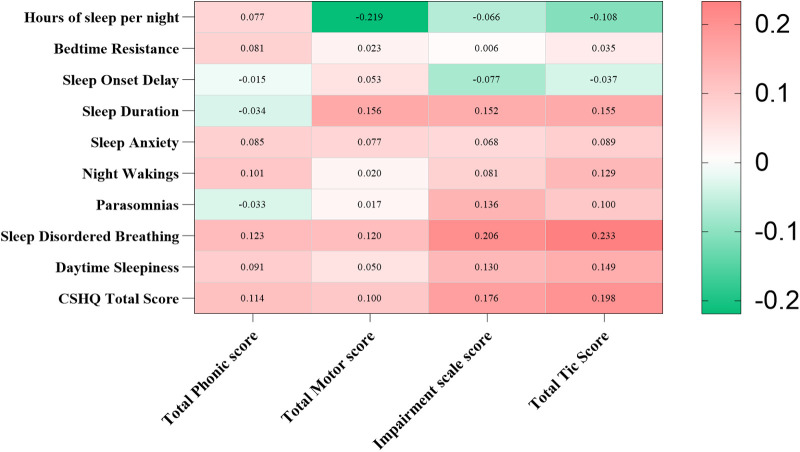
Correlation of YGTSS and CSHQ scores in children with tic disorder. YGTSS, Yale Global Tic Severity Scale; CSHQ, Children's Sleep Habits Questionnaire. Using Spearman’s rank correlation to analyze the relationship. Red represents positive correlation, while green represents negative correlation. *p < 0.05, **p < 0.01.

We identified a correlation between the YGTSS and CSHQ scores (Figure 3). In particular, hours of sleep per night were negatively correlated with total motor score (r = −0.219, *p* < 0.01), and sleep duration was positively correlated with total motor score, impairment scale score, and total tic score (r = 0.156, r = 0.152, r = 0.155, all *p* < 0.05). Sleep-disordered breathing, daytime sleepiness, and CSHQ total score were positively correlated with impairment scale score and total tic score (all *p* < 0.05).

## 4 Discussion

This study found no significant association between comorbid allergic diseases and the type of TD; however, a significant correlation was observed with the severity of TD. Moreover, these allergic conditions were strongly linked to sleep disturbances in children with TD, notably in relation to parasomnias, sleep-disordered breathing, and CSHQ scores. To the best of our knowledge, this is the first study to investigate the relationships among comorbid allergic disorders, symptom severity, and sleep in children with TD.

Baseline data indicated that TD children with allergic diseases exhibited significantly higher electronic screen exposure compared with their non-allergic counterparts. This observation may be attributable to the fact that airborne pollen—a common trigger of allergic diseases in northern China (26)—leads to a reduced outdoor activity among allergic children. Furthermore, the rate of parental allergy was significantly higher in the TD combined allergy group, which correlates with genetic susceptibility to allergic diseases. In cases where neither parent is allergic, the likelihood of the child developing allergies ranges between 5% and 16%. If one parent is allergic, this likelihood increases to 20%–40%, and if both parents are allergic, the risk exceeds 40%–60% (33).

This study found that comorbid allergies were significantly positively correlated with the severity of TD symptoms, a finding that aligns with previous research (34, 35). Liu et al. (35) used FACS to count immune cell subpopulations and observed that allergies increased the severity of TD through cellular immunity imbalance, particularly decreased levels of CD3, CD4, and CD4:CD8. However, our study found higher impairment scale scores and total tic scores in TD children with comorbid allergies, whereas Liu et al. detected that motor and vocal tic scores are higher. This variation may be attributed to differences in demographic profiles and sample sizes between the studies. Specifically, our study included children aged 4–14 years, whereas Liu et al.'s study focused on children aged 6–9 years. Notably, our findings revealed an increasing trend in both motor and vocal tic scores, a pattern that may become more pronounced with larger samples.

Neurotransmitter dysregulation may represent a critical factor underlying the co-occurrence of TD and allergic diseases. In particular, histamine plays a fundamental role in the development of several allergic conditions, such as AR and AA, by variably controlling T-helper lymphocytes (36). Gamma-aminobutyric acid (GABA) can suppress allergic responses in guinea pigs’ airways by activating GABA receptors (37). GABA is also capable of inhibiting the immune system, thus reducing allergic reactions (38). Concurrently, allergic sensitization has the potential to alter the lung expression of 5-hydroxytryptamine (5-HT) receptors in guinea pigs (39). Administration of 5-hydroxytryptophan (5-HTP), an antecedent to 5-HT, has been shown to mitigate allergic pulmonary inflammation and attenuate allergen-induced airway reactivity (40). These findings reveal a strong correlation between alterations in different neurotransmitters and the emergence of allergies.

Although elucidating the precise cellular and molecular basis of TD remains challenging, evidence indicates that cortico-striato-thalamocortical circuits, linking regions of the frontal cortex to subcortical structures, are involved (41). Within these neural pathways, signal transmission is modulated by several neurotransmitters, including dopamine, histamine, GABA, and 5-HT (42). Alterations in the brain's histamine modulation mechanism are acknowledged as contributing to the development of tics, and genetic research indicates that histamine imbalances can result in TD. Activation of histamine H3 receptors in the dorsal striatum has been shown to initiate repetitive behaviors in a TD mouse model (43). Mice deficient in histidine decarboxylase, an essential enzyme for histamine synthesis, exhibit persistent behavioral abnormalities, imbalanced dopamine levels, and altered indicators of cellular activity and synaptic communication within the striatum (43). Research has also revealed that people with TS exhibit heightened GABA levels in brain regions associated with the planning and choosing of movements (44). Another study suggests that elevated GABA levels may contribute to improved regulation of motor excitability in TS (45). Furthermore, the levels of 5-HT and 5-hydroxyindoleacetic acid may contribute to the development of TD, yet these results show no notable association with TD's intensity (46). In conclusion, neurotransmitter dysregulation in allergic conditions appears to be associated with the development of TD; however, the underlying mechanisms require further investigation.

In our study, comorbid allergies were significantly associated with sleep disturbances in children with TD, particularly parasomnias and sleep-disordered breathing. Sleep disorders are notably prevalent among TD patients (47). Approximaely65% of TD children have sleep disorders, including night waking, parasomnias, and sleep onset delay (48, 49). Findings from a nationwide case–control study indicated a higher likelihood of sleep disorders in TD patients combined with AR, aligning with our results (50). For an extended period, sleep disturbances associated with allergic diseases in TD children have not received adequate attention. However, a recent study involving more than 5,000 patients across 10 European countries has identified an association between insufficient sleep and respiratory as well as nasal symptoms (51). Additionally, it has been discovered that sleep disturbances play a mediating role in the link between AA, AR, and psychological distress in children (52). An increased eosinophil count in the peripheral blood correlates with a heightened severity of sleep disturbances (53), which explains the allergy–sleep correlation.

Our study demonstrated that the type of allergic disease was significantly associated with both TD symptom severity and sleep quality. In the combined FA group, TD symptoms and sleep were not significantly altered, possibly due to the occurrence of FA, predominantly between 1 and 3 years, which is associated with a longer time interval between the onset of TD (54). Furthermore, TD children with comorbid AC exhibited significantly higher total motor scores, whereas those with comorbid AR demonstrated significantly elevated impairment scale scores. This disparity may be attributable to the distinct symptomatic characteristics associated with AC and AR. Moreover, frequent eye blinking is commonly reported as an initial symptom in both AC and TD (55), which overlaps may lead to blinks due to AC being assessed in the motor score for TD, resulting in a higher total motor score.

In TD children with comorbid AR, the chronic nature of AR appears to affect TD, and behaviors such as frequent runny nose, sneezing, and nose picking have a serious impact on the children's ability to concentrate and interpersonal relationships (56, 57), which may explain the higher impairment scale score in this group. Unexpectedly, the data revealed that children with comorbid AD exhibited lower total motor scores, although no significant differences were observed in total tic scores. Currently, we are unable to provide a plausible explanation for this finding, warranting further investigation with an expanded sample size.

A study showed a positive correlation between the number of comorbid allergic conditions and the incidence of attention deficit–hyperactivity disorder (58). Unfortunately, the results of our study showed no statistical differences based on the number of allergic diseases. Although ADHD and TD are both common neurodevelopmental disorders, there are significant differences in their development. The differential impact of combining different numbers of allergic disorders in both needs to be further explored. Another study showed that controlling allergies is critical to improving the symptoms of TD (34). However, our study did not find a relevant effect of allergic disease control status on TD for the time being, which may be related to our inclusion of a small number of people with poorly controlled allergic diseases, and further follow-up of this outcome is needed based on subsequent expansion of the sample size.

This study has several limitations. Firstly, sleep information was mainly obtained from subjective parental questionnaires. Subjective methods offer various advantages, including non-invasive collection and low cost. However, the use of subjective sleep measures is considered to be affected by expectations, psychological influences, and responder bias (59). Polysomnography is frequently hailed as the “gold standard” for measuring sleep, gathering nocturnal physiological information such as brain function, ocular motion, cardiac rhythm, and blood oxygen concentrations (60). Variations may be observed between the subjective sleep reports and objective sleep measures (61). Therefore, there is a need to use a combination of subjective and objective measures to better understand sleep in children with TD. Secondly, this study lacked relevant cytokine assays and failed to further explore the relationship between allergic diseases and TD from a mechanistic perspective. This is also a part of this study that needs to be improved in the next step. Thirdly, the correlation analysis revealed that the severity of TD was associated with sleep disturbances, and the inclusion of a control group comprising children with allergic diseases but without TD could have provided a clearer understanding of the relationship between allergies and sleep in children with TD. Unfortunately, this study did not collect data on children with allergies only, indicating an area for improvement in future research. Fourthly, the OSA diagnosis, family economic situation, and parents’ education level of the two groups of children were not collected, and the impact of the above factors on the outcomes needs to be considered in future studies. Finally, this study utilized a cross-sectional retrospective design to primarily investigate the association between combined allergic diseases, TD symptoms, and sleep. Although a bidirectional relationship between sleep and TD may exist, the findings of this study do not permit causal inferences. Future research should implement prospective cohort designs to elucidate the causal relationship between these variables further.

## 5 Conclusions

In summary, this study found that comorbid allergies had a notable impact on TD symptom severity and sleep. Among allergic diseases, allergic rhinitis and allergic conjunctivitis had the most significant effect on TD. We should be mindful to focus on the possible effects of comorbid allergy on symptoms and sleep in children with TD.

## Data Availability

The original contributions presented in the study are included in the article/Supplementary Material, further inquiries can be directed to the corresponding author.
