# Supplementary material for: Symptoms and sleep characteristics of tic disorder children with allergic diseases: a case–control study
Source: Front Pediatr. 2025 Sep 30;13:1573463. doi: 10.3389/fped.2025.1573463 (PMC12518102; doi:10.3389/fped.2025.1573463)
Supplement: Supplementary file 1 [file Table1.docx]

**Supplement Table 1: Types , YGTSS and CSHQ scores between different number of combine allergic diseases in TD children**

|  |  | **TD+ 1 Allergy group** | **TD+ 2 Allergy group** | **TD+ 3 Allergy group** | **TD+≥4 Allergy group** | **Statistics** |
| --- | --- | --- | --- | --- | --- | --- |
| Types of TD  n（%） | PTD | 54 | 28 | 13 | 3 | *χ*²=5.629*, p*=0.567 |
|  | CTD | 16 | 7 | 7 | 0 |  |
|  | TS | 17 | 12 | 9 | 2 |  |
| YGTSS  （Mean ± SD ） | Total Phonic score | 4.66 ± 4.62 | 5.04 ± 4.80 | 4.48 ± 4.43 | 6.00 ± 4.30 | *H*=0.733*, p*=0.865 |
|  | Total Motor score | 9.41 ± 3.99 | 8.70 ± 4.14 | 9.48 ± 3.61 | 11.40 ± 3.85 | *H*=2.009*, p*=0.571 |
|  | Impairment scale score | 14.71± 7.13 | 14.68 ± 6.87 | 15.00 ± 6.55 | 14.00 ± 5.48 | *H*=0.095*, p*=0.992 |
|  | Total Tic Score | 28.78 ± 10.34 | 28.43 ± 10.16 | 28.97 ± 9.57 | 31.40 ± 9.24 | *H*=0.775*, p*=0.855 |
| CSHQ  （Mean ± SD ） | Hours of sleep per night | 9.44 ± 0.82 | 9.36 ± 0.76 | 9.48 ± 0.69 | 9.80± 1.10 | *H*=0.898*, p*=0.826 |
|  | Bedtime Resistance | 10.95 ± 2.98 | 10.89 ± 3.28 | 11.83 ± 2.77 | 9.60 ± 3.21 | *H*=3.276*, p*=0.351 |
|  | Sleep Onset Delay | 1.51 ± 0.66 | 1.72 ± 0.71 | 1.41 ± 0.50 | 1.60 ± 0.89 | *H*=4.317*, p*=0.229 |
|  | Sleep Duration | 4.32 ± 1.43 | 4.36 ± 1.44 | 3.66 ± 1.11 | 4.40 ± 1.52 | *H*=6.596*, p*=0.086 |
|  | Sleep Anxiety | 7.20 ± 2.26 | 7.21 ± 2.41 | 7.93 ± 2.00 | 5.80 ± 2.17 | *H*=5.144*, p*=0.162 |
|  | Night Wakings | 3.78 ± 1.08 | 3.83 ± 1.24 | 3.97 ± 1.32 | 3.80 ± 0.84 | *H*=0.355*, p*=0.949 |
|  | Parasomnias | 8.86 ± 1.76 | 9.11 ± 1.70 | 8.69 ± 2.02 | 8.80 ± 2.05 | *H*=2.058*, p*=0.560 |
|  | Sleep Disordered Breathing | 3.69 ± 0.93 | 3.62 ± 0.85 | 3.59 ± 0.82 | 3.80 ± 0.84 | *H*=0.744*, p*=0.863 |
|  | Daytime Sleepiness | 13.44 ± 3.29 | 13.43 ± 3.11 | 13.00 ± 3.05 | 13.60 ± 3.36 | *H*=0.722*, p*=0.868 |
|  | Total Score | 53.75 ± 8.69 | 54.17 ± 8.16 | 54.07 ± 7.04 | 51.40 ± 9.81 | *H*=0.434*, p*=0.933 |

TD: Tic disorder; YGTSS: Yale Global Tic Severity Scale; CSHQ: Children’s Sleep Habits Questionnaire. Comparisons between groups were made using Kruskal-Wallis H test.
