# Supplementary material for: Symptoms and sleep characteristics of tic disorder children with allergic diseases: a case–control study
Source: Front Pediatr. 2025 Sep 30;13:1573463. doi: 10.3389/fped.2025.1573463 (PMC12518102; doi:10.3389/fped.2025.1573463)
Supplement: Supplementary file 2 [file Table2.docx]

**Supplement Table 2: Effect of combined Food Allergies on types, YGTSS and CSHQ scores in TD children**

|  |  | **TD+ Food**  **Allergy group** | **TD+ No Food Allergy group** | **Statistics** |
| --- | --- | --- | --- | --- |
| Types of TD  n（%） | PTD | 21 | 122 | *χ*²=4.983*, p*=0.083 |
|  | CTD | 7 | 42 |  |
|  | TS | 14 | 36 |  |
| YGTSS  （Mean ± SD ） | Total Phonic score | 5.69 ± 4.83 | 4.32 ± 4.70 | *Z*=-1.816, *p*=0.069 |
|  | Total Motor score | 9.02 ± 4.03 | 9.27 ± 3.90 | *Z*=-0.203, *p*=0.839 |
|  | Impairment scale score | 14.40 ± 6.07 | 13.55 ± 6.57 | *Z*=-0.951, *p*=0.342 |
|  | Total Tic Score | 29.12 ± 9.74 | 27.14 ± 9.74 | *Z*=-1.339, *p*=0.181 |
| CSHQ  （Mean ± SD ） | Hours of sleep per night | 9.50 ± 0.83 | 9.45 ± 0.81 | *Z*=-0.071, *p*=0.943 |
|  | Bedtime Resistance | 11.29 ± 3.01 | 10.80 ± 3.08 | *Z*=-0.960, *p*=0.337 |
|  | Sleep Onset Delay | 1.52 ± 0.63 | 1.52 ± 0.66 | *Z*=-0.225, *p*=0.822 |
|  | Sleep Duration | 4.26 ± 1.48 | 4.19 ± 1.37 | *Z*=-0.111, *p*=0.912 |
|  | Sleep Anxiety | 7.40 ± 2.25 | 7.18 ± 2.24 | *Z*=-0.707, *p*=0.479 |
|  | Night Wakings | 3.98 ± 1.33 | 3.72 ± 1.04 | *Z*=-1.281, *p*=0.200 |
|  | Parasomnias | 8.93 ± 1.66 | 8.69 ± 1.72 | *Z*=-1.037, *p*=0.300 |
|  | Sleep Disordered Breathing | 3.57 ± 0.83 | 3.58 ± 0.86 | *Z*=-0.019, *p*=0.984 |
|  | Daytime Sleepiness | 13.88 ± 3.18 | 13.07 ± 3.01 | *Z*=-1.466, *p*=0.143 |
|  | Total Score | 54.83 ± 7.74 | 52.73 ± 7.93 | *Z*=-1.841, *p*=0.066 |

TD: Tic disorder; YGTSS: Yale Global Tic Severity Scale; PTD: Provisional tic disorders; CTD: Chronic motor or vocal tic disorders; TS: Tourette's syndrome; CSHQ: Children’s Sleep Habits Questionnaire. Comparisons between groups were using Mann-Whitney U test.
