# Supplementary material for: Symptoms and sleep characteristics of tic disorder children with allergic diseases: a case–control study
Source: Front Pediatr. 2025 Sep 30;13:1573463. doi: 10.3389/fped.2025.1573463 (PMC12518102; doi:10.3389/fped.2025.1573463)
Supplement: Supplementary file 3 [file Table3.docx]

**Supplement Table 3: Effect of combined** **Atopic dermatitis on types, YGTSS and CSHQ scores in TD children**

|  |  | **TD+ Atopic**  **dermatitis group** | **TD+ No Atopic**  **dermatitis group** | **Statistics** |
| --- | --- | --- | --- | --- |
| Types of TD  n（%） | PTD | 38 | 105 | *χ*²=1.751*, p*=0.417 |
|  | CTD | 11 | 38 |  |
|  | TS | 17 | 33 |  |
| YGTSS  （Mean ± SD ） | Total Phonic score | 4.82 ± 4.30 | 4.46 ± 4.91 | *Z*=-0.821, *p*=0.412 |
|  | Total Motor score | 8.52 ± 3.75 | 9.49± 3.95 | *Z*=-2.037, *p*=0.042* |
|  | Impairment scale score | 14.47 ± 6.91 | 13.41 ± 6.30 | *Z*=-1.001, *p*=0.317 |
|  | Total Tic Score | 27.80 ± 9.92 | 27.36 ± 9.71 | *Z*=-0.185, *p*=0.853 |
| CSHQ  （Mean ± SD ） | Hours of sleep per night | 9.53 ± 0.73 | 9.43 ± 0.83 | *Z*=-0.761, *p*=0.447 |
|  | Bedtime Resistance | 11.32 ± 3.10 | 10.72 ± 3.05 | *Z*=-1.414, *p*=0.157 |
|  | Sleep Onset Delay | 1.52 ± 0.61 | 1.52 ± 0.68 | *Z*=-0.251, *p*=0.802 |
|  | Sleep Duration | 3.88 ± 1.27 | 4.32 ± 1.41 | *Z*=-2.330, *p*=0.020* |
|  | Sleep Anxiety | 7.38 ± 2.13 | 7.15 ± 2.28 | *Z*=-0.799, *p*=0.424 |
|  | Night Wakings | 3.92 ± 1.28 | 3.70 ± 1.02 | *Z*=-1.052, *p*=0.293 |
|  | Parasomnias | 8.85 ± 1.85 | 8.69 ± 1.65 | *Z*=-0.449, *p*=0.653 |
|  | Sleep Disordered Breathing | 3.56 ± 0.77 | 3.58 ± 0.88 | *Z*=-0.125, *p*=0.900 |
|  | Daytime Sleepiness | 13.20 ± 2.98 | 13.21 ± 3.09 | *Z*=-0.034, *p*=0.973 |
|  | Total Score | 53.62 ± 8.09 | 52.89 ± 7.87 | *Z*=-0.637, *p*=0.524 |

YGTSS: Yale Global Tic Severity Scale; CSHQ: Children’s Sleep Habits Questionnaire.; TD: Tic disorder; PTD: Provisional tic disorders; CTD: Chronic motor or vocal tic disorders; TS: Tourette's syndrome. *: there is a statistical difference between the two groups, *p*< 0.05.
