# Supplementary material for: Symptoms and sleep characteristics of tic disorder children with allergic diseases: a case–control study
Source: Front Pediatr. 2025 Sep 30;13:1573463. doi: 10.3389/fped.2025.1573463 (PMC12518102; doi:10.3389/fped.2025.1573463)
Supplement: Supplementary file 4 [file Table4.docx]

**Supplement Table 4: Effect of combined Allergic rhinitis on types, YGTSS and CSHQ scores in TD children**

|  |  | **TD+ Allergy rhinitis group** | **TD+ No Allergy**  **rhinitis group** | **Statistics** |
| --- | --- | --- | --- | --- |
| Types of TD  n（%） | PTD | 74 | 69 | *χ*²=5.873*, p*=0.053 |
|  | CTD | 24 | 25 |  |
|  | TS | 35 | 15 |  |
| YGTSS  （Mean ± SD ） | Total Phonic score | 4.95 ± 4.56 | 4.08 ± 4.94 | *Z*=-1.708, *p*=0.088 |
|  | Total Motor score | 9.34 ± 3.92 | 9.08 ± 3.92 | *Z*=-1.004, *p*=0.316 |
|  | Impairment scale score | 14.77 ± 6.85 | 12.39 ± 5.76 | *Z*=-2.842, *p*=0.004** |
|  | Total Tic Score | 29.06 ± 10.04 | 25.55 ± 9.06 | *Z*=-2.925, *p*=0.003** |
| CSHQ  （Mean ± SD ） | Hours of sleep per night | 9.42 ± 0.76 | 9.50 ± 0.86 | *Z*=-0.699, *p*=0.485 |
|  | Bedtime Resistance | 11.02 ± 3.01 | 10.72 ± 3.14 | *Z*=-0.591, *p*=0.555 |
|  | Sleep Onset Delay | 1.57 ± 0.68 | 1.45 ± 0.63 | *Z*=-1.452, *p*=0.146 |
|  | Sleep Duration | 4.23 ± 1.40 | 4.17 ± 1.38 | *Z*=-0.407, *p*=0.684 |
|  | Sleep Anxiety | 7.29 ± 2.32 | 7.12 ± 2.15 | *Z*=-0.607, *p*=0.544 |
|  | Night Wakings | 3.77 ± 1.11 | 3.76 ± 1.08 | *Z*=-0.080, *p*=0.936 |
|  | Parasomnias | 8.84 ± 1.80 | 8.60 ± 1.59 | *Z*=-0.868, *p*=0.386 |
|  | Sleep Disordered Breathing | 3.72 ± 0.91 | 3.39 ± 0.75 | *Z*=-3.354, *p*<0.001*** |
|  | Daytime Sleepiness | 13.44 ± 3.16 | 12.92 ± 2.91 | *Z*=-1.253, *p*=0.210 |
|  | Total Score | 53.89 ± 7.99 | 52.12 ± 7.76 | *Z*=-1.906, *p*=0.057 |

YGTSS: Yale Global Tic Severity Scale; CSHQ: Children’s Sleep Habits Questionnaire; TD: Tic disorder; PTD: Provisional tic disorders; CTD: Chronic motor or vocal tic disorders; TS: Tourette's syndrome. **: there is a statistical difference between the two groups, *p*< 0.01; ***: there is a statistical difference between the two groups, *p*< 0.001.
