# Supplementary material for: Symptoms and sleep characteristics of tic disorder children with allergic diseases: a case–control study
Source: Front Pediatr. 2025 Sep 30;13:1573463. doi: 10.3389/fped.2025.1573463 (PMC12518102; doi:10.3389/fped.2025.1573463)
Supplement: Supplementary file 5 [file Table5.docx]

**Supplement Table 5: Effect of combined Allergic conjunctivitis on types, YGTSS and CSHQ scores in TD children**

|  |  | **TD+ Allergic conjunctivitis group** | **TD+ No Allergic conjunctivitis group** | **Statistics** |
| --- | --- | --- | --- | --- |
| Types of TD  n（%） | PTD | 23 | 120 | *χ*²=0.172*, p*=0.918 |
|  | CTD | 7 | 42 |  |
|  | TS | 7 | 43 |  |
| YGTSS  （Mean ± SD ） | Total Phonic score | 3.32 ± 4.05 | 4.78 ± 4.83 | *Z*=-1.608, *p*=0.108 |
|  | Total Motor score | 10.86 ± 3.92 | 8.93 ± 3.85 | *Z*=-3.338, *p*<0.001*** |
|  | Impairment scale score | 15.14 ± 6.51 | 13.44 ± 6.46 | *Z*=-1.875, *p*=0.061 |
|  | Total Tic Score | 29.32 ± 8.08 | 25.15 ± 10.00 | *Z*=-1.990, *p*=0.047* |
| CSHQ  （Mean ± SD ） | Hours of sleep per night | 9.43 ± 0.96 | 9.46 ± 0.78 | *Z*=-0.424, *p*=0.671 |
|  | Bedtime Resistance | 10.92 ± 3.04 | 10.88 ± 3.08 | *Z*=-0.072, *p*=0.943 |
|  | Sleep Onset Delay | 1.59 ± 0.69 | 1.50 ± 0.65 | *Z*=-0.814, *p*=0.415 |
|  | Sleep Duration | 3.92 ± 1.14 | 4.25 ± 1.43 | *Z*=-1.058, *p*=0.290 |
|  | Sleep Anxiety | 7.30 ± 2.37 | 7.20 ± 2.22 | *Z*=-0.190, *p*=0.850 |
|  | Night Wakings | 3.92 ± 1.14 | 3.74 ± 1.09 | *Z*=-0.967, *p*=0.334 |
|  | Parasomnias | 9.05 ± 2.17 | 8.67 ± 1.61 | *Z*=-0.508, *p*=0.612 |
|  | Sleep Disordered Breathing | 3.70 ± 0.91 | 3.55 ± 0.84 | *Z*=-1.027, *p*=0.304 |
|  | Daytime Sleepiness | 12.19 ± 2.85 | 13.39 ± 3.06 | *Z*=-2.206, *p*=0.027* |
|  | Total Score | 52.59 ± 7.61 | 53.18 ± 7.99 | *Z*=-0.287, *p*=0.774 |

YGTSS: Yale Global Tic Severity Scale; CSHQ: Children’s Sleep Habits Questionnaire; TD: Tic disorder; PTD: Provisional tic disorders; CTD: Chronic motor or vocal tic disorders; TS: Tourette's syndrome. *: there is a statistical difference between the two groups, *p*< 0.05; ***: there is a statistical difference between the two groups, *p*< 0.001.
