# Supplementary material for: Symptoms and sleep characteristics of tic disorder children with allergic diseases: a case–control study
Source: Front Pediatr. 2025 Sep 30;13:1573463. doi: 10.3389/fped.2025.1573463 (PMC12518102; doi:10.3389/fped.2025.1573463)
Supplement: Supplementary file 6 [file Table6.docx]

**Supplement table 6: Effect of allergic disease control on types, YGTSS and CSHQ scores in TD children**

|  |  | **TD+ Allergic complete controlled group**  **N=102** | **TD+ Allergic partial**  **controlled group**  **N=54** | **TD+ Allergic poorly**  **controlled group**  **N=12** | **Statistics** |
| --- | --- | --- | --- | --- | --- |
| Types of TD  n（%） | PTD | 65 | 26 | 7 | *χ*²=4.821*, p*=0.306 |
|  | CTD | 16 | 13 | 1 |  |
|  | TS | 21 | 15 | 4 |  |
| YGTSS  （Mean ± SD ） | Total Phonic score | 4.38 ± 4.61 | 5.13 ± 4.67 | 6.50 ± 4.03 | *Z*=3.107, *p*=0.211 |
|  | Total Motor score | 9.36 ± 4.01 | 9.33 ± 3.78 | 8.42 ± 4.58 | *Z*=0.286, *p*=0.867 |
|  | Impairment scale score | 14.95 ± 6.47 | 14.63 ± 7.94 | 13.33 ± 4.92 | *Z*=0.817, *p*=0.665 |
|  | Total Tic Score | 28.70 ± 9.51 | 29.09 ± 11.27 | 28.25 ± 9.55 | *Z*=0.016, *p*=0.992 |
| CSHQ  （Mean ± SD ） | Hours of sleep per night | 9.36 ± 0.79 | 9.59 ± 0.79 | 9.33 ± 0.65 | *H*=3.757, *p*=0.153 |
|  | Bedtime Resistance | 11.26 ± 2.89 | 10.93 ± 3.27 | 9.75 ± 3.14 | *H*=2.530, *p*=0.282 |
|  | Sleep Onset Delay | 1.52 ± 0.66 | 1.59 ± 0.66 | 1.67 ± 0.78 | *H*=0.787, *p*=0.675 |
|  | Sleep Duration | 4.30 ± 1.45 | 3.93 ± 1.21 | 4.83 ±1.59 | *H*=4.199 *p*=0.123 |
|  | Sleep Anxiety | 7.28 ± 2.13 | 7.54 ± 2.53 | 6.17 ± 2.08 | *H*=3.635, *p*=0.162 |
|  | Night Wakings | 3.81 ± 1.17 | 3.89 ± 1.14 | 3.67 ± 1.23 | *H*=0.675, *p*=0.714 |
|  | Parasomnias | 8.94 ± 1.75 | 8.85 ± 1.85 | 8.75 ± 2.01 | *H*=0.487, *p*=0.784 |
|  | Sleep Disordered Breathing | 3.55 ± 0.77 | 3.80 ± 1.02 | 3.92 ± 1.08 | *H*=2.859, *p*=0.239 |
|  | Daytime Sleepiness | 13.45 ± 3.21 | 12.87 ± 3.14 | 14.83± 2.69 | *H*=4.109, *p*=0.128 |
|  | Total Score | 54.13 ± 8.19 | 53.39 ± 8.73 | 53.58 ± 7.01 | *H*=0.090, *p*=0.956 |

TD: Tic disorder; YGTSS: Yale Global Tic Severity Scale; CSHQ: Children’s Sleep Habits Questionnaire. Comparisons between groups were made using Kruskal-Wallis H test.
